# Predictors of Presence of and Search for Meaning in Life among Omani Students during the COVID-19 Pandemic: A Cross-sectional Study

**DOI:** 10.1192/j.eurpsy.2025.385

**Published:** 2025-08-26

**Authors:** T. Al-Mahrouqi, R. Al-Obeidani, M. Elsherif, M. Al Shehi, M. Al-Mukhaini, S. Al-Huseini, F. Jahan, N. Al Balushi, M. Al-Alawi

**Affiliations:** 1 Oman Medical Speciality Board; 2 Sultan Qaboos University Hospital, Muscat, Oman; 3 Ministry of Health, Kuwait, Kuwait; 4 National University of Science and Technology, Muscat, Oman

## Abstract

**Introduction:**

While various fields and work areas have been impacted due to COVID-19, undergraduate students appear to have compounded stress. We sought to investigate the variables that predict meaning in life for Omani college students during the COVID-19 pandemic.

**Objectives:**

This study investigated the personal and academic factors associated with the presence and search for meaning in life among college students in Oman.

**Methods:**

A cross-sectional study was conducted in April 2021. A self-reported survey comprising the Meaning in Life Questionnaire (MLQ) and a sociodemographic questionnaire was completed by 970 students at the National University of Science and Technology in Oman. We used multiple linear regression to explore the independent predictors.

**Results:**

Compared with engineering students, medical students were found to have a higher degree of both the presence of meaning in life as well as the search for meaning in life (p-value 0.001), and with each advancing academic year, the presence of meaning in life was found to be lower (p-value = 0.002). Students with chronic physical disease had a lower degree of presence of meaning in life and a lower degree of search for meaning in life (p = 0.001) compared with those without chronic disease. In addition, mental illness was associated with a lower degree of presence of meaning in life (p-value 0.001) and financial strain was associated with a lower degree of presence of meaning in life (p-value = 0.001).

**Conclusions:**

In conclusion, no prior research demonstrated higher levels of meaning in life among medicine major students compared to those in engineering or pharmacy majors. Moreover, other academic, socio-economic, and health-related factors correlated with individuals’ sense of meaning & search in life. Therefore, psychologists and psychiatrists should consider these diverse factors when designing interventions to support individuals in exploring and enhancing their meaning in life, considering their unique needs and contexts.

**Disclosure of Interest:**

None Declared

